# A Rare Case of Nasopharyngeal Pleomorphic Adenoma

**DOI:** 10.1155/2013/712873

**Published:** 2013-04-30

**Authors:** S. Berrettini, S. Fortunato, A. De Vito, L. Bruschini

**Affiliations:** Otolaringology, Audiology and Phoniatric Unit, University of Pisa, Via Paradisa 2, 56126 Pisa, Italy

## Abstract

Salivary gland tumors are rare. The majority of these tumors are benign and about 70% are pleomorphic adenomas (PA). Nasopharynx is an unusual site for the PA tumor. Only six cases are presented in the literature from 1990 to 2011. The diagnosis of this disease is linked to the sum of imaging tests, clinical and histological study of the mass of the above. The radiologic features of noninvasion of surrounding structures give the first clue to the benign lesion. From the review of the literature and our experience is identified as the gold standard in surgical treatment for this condition. Thanks to the endoscopic surgery, we can perform a complete resection of the lesion without damaging adjacent structures. We presented a case of pleomorphic adenoma of nasopharynx with literature review.

## 1. Introduction

Salivary gland tumors are rare, with less than 5% of all head and neck neoplasms forming in the salivary gland, commonly in the parotid [1].

The majority of these tumors are benign and about 70% are pleomorphic adenomas (PA). It mainly occurs in the parotid gland and submandibular gland. If the tumor occurs in the minor salivary glands, the most common site is the palate, [2] but this tumor can also occur in other sites including the upper lip, cheek, pharynx, floor of the mouth, larynx, and trachea. The first site of origin in nasopharynx is extremely rare [3, 4].

The aim of this paper is to present a case of PA of the nasopharynx. Moreover, we report a literature review that reveals only six cases like the present one.

## 2. Case Report

A 51-year-old woman was referred to us with a progressive nasal obstruction associated left aural fullness and pain with a middle conductive hearing loss. The symptoms arose two years before. In home hospital, the patient has been already evaluated with a Magnetic Resonance.

**Figure 1:**
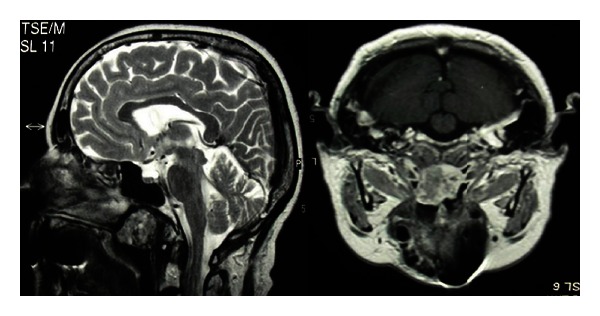
Magnetic Resonance. A mass connected to the lateral wall of nasopharynx space is identified. The lesion does not infiltrate the adjacent tissue and had lobed margins.

These imaging tests showed a mass connected to the lateral wall of nasopharynx space. The lesion does not infiltrate the adjacent tissue and had lobed margins. The radiologist retained that the images were assimilable to pleomorphic adenoma (Figure 1). The chemoradiotherapy was the first choice treatment but the tumor did not drop and the symptoms worsened.

In our hospital, on endoscopic examination the lesion appears as a soft whitish mass of 3 × 4 × 3 cm; it took up the entire nasopharynx.

**Figure 2:**
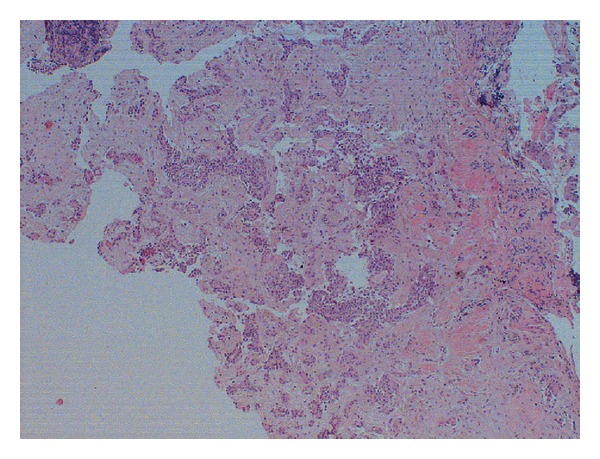
Biopsy fragment characterized by the mixture of elements myoepithelial and stromal areas of mixocondoide appearance (hematoxylin-eosin 40x).

**Figure 3:**
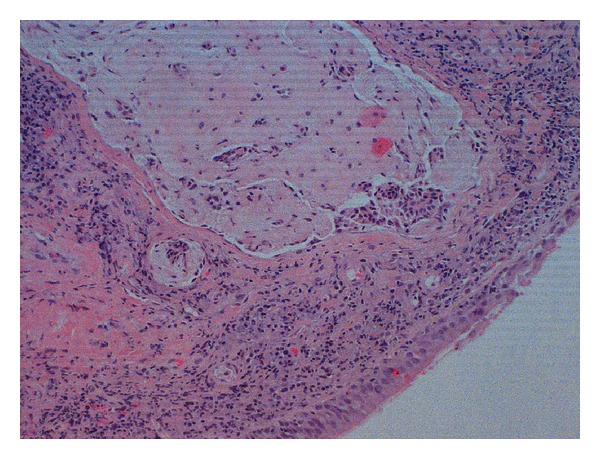
Small lump of chondroid appearance comprising aggregates of myoepithelial elements, covered by normal mucous membrane of respiratory type (bottom right) (hematoxylin-eosin 100x).

We performed a biopsy in order to obtain a definitive histological evaluations. The result confirmed the diagnosis of pleomorphic adenoma with mixture of myoepithelial elements and stromal areas of aspect mixocondoide (Figures 2 and 3).

**Figure 4:**
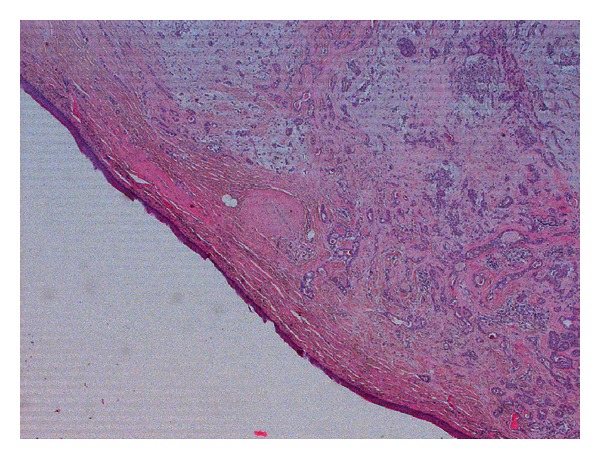
Entire section of the lesion (hematoxylin-eosin 25x).

We decided to perform a complete excision of the lesion, by endoscopic transnasal approach. The histological section is represented in Figure 4. We advocated use of endoscope to avoid injury of Eustachian tube. After surgery the Eustachian tube function improved and aural fullness disappeared after 2-3 days together with hearing loss.

After the surgery, we removed the whole lesion and the patient reported an improvement in symptoms and after 6 months the nasopharynx was disease-free.

## 3. Discussion and Literature Review

Nasopharynx is an unusual site for the PA tumor. Only six cases like the present one are described in international literature from 1990 to 2011.

The diagnosis of PA is simple. But when the PA grows in unusual site like the pharyngeal cavity, it can be mistaken for malignant neoplasms. Furthermore, symptoms such as nasal obstruction, epistaxis, and ear pain are related to nasopharyngeal carcinoma. The PA induced only nasal obstruction. PA should be differentially diagnosed from various other tumors, such as angiofibroma, hamartoma, epidermoid cyst, hemangioma, vascular malformations, nasopharyngeal carcinoma, and nonepithelial tumors; therefore, an incisional biopsy is always a crucial step in the management of these lesions. Although histology is sometimes hard to evaluate, PA is characterized by epithelial tissue mixed with tissues of myxoid, mucoid, or chondroid appearance. Occasionally, pleomorphic adenomas are composed almost entirely of epithelial cells with few or no stromatic tissue. This can lead to misdiagnosis as a carcinoma.

The PA is always treated by the surgical exeresis. Although PA of the nasopharynx is a highly curable disease, the surgical approach is not simple and there is the possibility of postoperative complications, such as dysfunctions of the Eustachian tube. For this reason the choice of surgical technique is essential to avoid postoperative complications, but at the same time it is able to dominate the mass in its entirety [3]. Various surgical approaches have been developed such as the transpalatal, transmaxillary, transmandibular, and transpterygoid, but the external approaches may lead to postoperative morbility [3]. With the development of endoscopic techniques complications were reduced considerably and you get a complete exposure of the lesion.

Roh et al. treated a similar case with surgical approach using the endoscope. Also Lee et al. used the endoscopic approach with excellent result. Thakur et al. were the only ones to utilize the FNAB for first and erroneous diagnosis of carcinoma. The nasopharynx “malignant tumor” was treated with a radiochemo therapy without any result. Then, an incisional biopsy was performed and was found to be suggestive of a pleomorphic adenoma. A surgical intervention was planned, and tumor was excised by transpalatal approach under general anesthesia.

## 4. Conclusions

PA of the nasopharynx is a very rare disease. The diagnosis of this disease is linked to the sum of imaging tests, clinical and histological study of the mass of the above. The radiologic features of noninvasion of surrounding structures give the first clue to the benign lesion. The appearance of the lesion, not bleeding and multilobulated, gives us the second clue. Finally, histological examination gives us the final confirmation of the diagnosis.

From the review of the literature and our experience is identified as the gold standard in surgical treatment for this condition. Thanks to the endoscopic surgery, we can perform a complete resection of the lesion without damaging adjacent structures. In some articles considering radiotherapy but in all concludes that this last do not have any action on the symptoms and the size of the lesion.
